# Personalized Monitoring Model for Electrocardiogram Signals: Diagnostic Accuracy Study

**DOI:** 10.2196/24388

**Published:** 2020-12-29

**Authors:** Rado Kotorov, Lianhua Chi, Min Shen

**Affiliations:** 1 Trendalyze Inc Newark, NJ United States; 2 La Trobe University Bundoora Australia

**Keywords:** COVID-19, personalized monitoring model, ECG, time series, motif discovery, monitoring, heart disease, electrocardiogram

## Abstract

**Background:**

Due to the COVID-19 pandemic, the demand for remote electrocardiogram (ECG) monitoring has increased drastically in an attempt to prevent the spread of the virus and keep vulnerable individuals with less severe cases out of hospitals. Enabling clinicians to set up remote patient ECG monitoring easily and determining how to classify the ECG signals accurately so relevant alerts are sent in a timely fashion is an urgent problem to be addressed for remote patient monitoring (RPM) to be adopted widely. Hence, a new technique is required to enable routine and widespread use of RPM, as is needed due to COVID-19.

**Objective:**

The primary aim of this research is to create a robust and easy-to-use solution for personalized ECG monitoring in real-world settings that is precise, easily configurable, and understandable by clinicians.

**Methods:**

In this paper, we propose a Personalized Monitoring Model (PMM) for ECG data based on motif discovery. Motif discovery finds meaningful or frequently recurring patterns in patient ECG readings. The main strategy is to use motif discovery to extract a small sample of personalized motifs for each individual patient and then use these motifs to predict abnormalities in real-time readings of that patient using an artificial logical network configured by a physician.

**Results:**

Our approach was tested on 30 minutes of ECG readings from 32 patients. The average diagnostic accuracy of the PMM was always above 90% and reached 100% for some parameters, compared to 80% accuracy for the Generalized Monitoring Models (GMM). Regardless of parameter settings, PMM training models were generated within 3-4 minutes, compared to 1 hour (or longer, with increasing amounts of training data) for the GMM.

**Conclusions:**

Our proposed PMM almost eliminates many of the training and small sample issues associated with GMMs. It also addresses accuracy and computational cost issues of the GMM, caused by the uniqueness of heartbeats and training issues. In addition, it addresses the fact that doctors and nurses typically do not have data science training and the skills needed to configure, understand, and even trust existing black box machine learning models.

## Introduction

### Background

An electrocardiogram (ECG) is a medical test that records the electrical activities of the heart. It is widely used by medical practitioners for diagnosing cardiac conditions by detecting irregular heart rhythms and abnormalities [1]. In some cases, arrhythmic heartbeats can be lethal and the risk of sudden death is significant without remote patient monitoring (RPM) [2]. Therefore, it is highly desirable for patients to have an efficient ECG remote monitoring system that can identify life-threatening situations and send alerts to their health care providers [3]. Lately, the demand for remote ECG monitoring has increased drastically because of the COVID-19 pandemic. To prevent the spread of the virus and keep individuals with less severe cases out of hospitals, more patients are having heart disease diagnosed and monitored remotely, while at home. The accuracy of the ECG signal classifier is becoming more important because false alarms can overwhelm the system. Therefore, classifying the ECG signals accurately and sending alerts to health care professionals in a timely fashion are urgent problems that need to be addressed.

The classification of ECG signals is an extremely challenging problem as there are no defined optimal classification rules. Many researchers have focused on developing different machine learning models, such as Bayesian framework [4], random forest [5], gradient boosting [6], ensemble boosting [7], and support vector machine [8], among others, and achieved relatively high accuracy. To extract different features for models, various techniques were proposed, such as principal component analysis [9-12], wavelet transform [13], and filter banks [14]. Deep learning methods such as deep neural networks [15], convolutional neural networks [16-20], and recurrent neural networks [21] are also applied extensively to classification problems. Deep learning can be a powerful tool for solving cognitive problems [22], but accurately training such models requires large amounts of labeled data [23]. For clinical applications, due to limited patient contact, variation in medical care, and privacy issues, getting a large amount of high-quality data can be very challenging [24], and the efficacy of deep learning methods can be greatly affected by the lack of training data [25-29]. According to Chen et al’s [30] investigation of the training time of deep learning and machine learning methods, deep learning requires a longer training time compared to conventional machine learning algorithms. In addition, building and maintaining the computational infrastructure required for deep learning can be too costly for small health care organizations to implement. Thus, a less computationally expensive method is needed to effectively resolve the issue.

Another limitation of deep learning methods is that the models are not able to capture the individuality of the ECG features and patterns [31]. Most of the deep learning models are generalized models and are not able to be built to the individual level due to a lack of data [26]. However, each patient has unique heartbeats and the waveforms can be completely different on an individual level. Hence, accuracy might be an issue for these models when using real-time data. Traditional machine learning methods usually require more effort related to data preprocessing and feature engineering compared to deep learning models [29]. In addition, they tend to be like black boxes to medical practitioners without a data science background. The lack of interpretability can hinder health care providers’ decision making process and communication with patients.

In this paper, we propose a Personalized Monitoring Model (PMM) for ECG monitoring based on motif discovery to address the abovementioned challenges. Motif discovery is a method for analyzing large amounts of time series data. In the health care domain, it has been used for trend analysis and data summarization [32]. Motifs are defined as frequently recurring patterns in certain time series [33]. In a motif discovery process, a similarity search is conducted based on a certain similarity threshold to detect and locate previously defined patterns. In a similarity search, the distances between time series subsequences are calculated, which indicates how similar two subsequences are.

### Objectives

The primary aim of this research is to create a robust and easy-to-configure solution for monitoring ECG signals in real-world settings. We developed a technique for building personalized prediction models to address the limitations of generalized models [31]. The main strategy of the model is to extract personalized motifs for each patient and use the motifs to predict the rest of the readings of that patient using an artificial logical network. By performing a systematic analysis and evaluation, we will investigate the hypothesis that the proposed PMM is more accurate and efficient than generalized models. In most cases, doctors and nurses do not have a data science background and the existing machine learning models might be difficult to configure. Hence, a new technique will be required as RPM becomes more common, as has occurred due to the COVID-19 pandemic. The main goal is to develop a technique that allows doctors, nurses, and other medical practitioners to easily configure a personalized model for RPM. The proposed model can be easily understood and configured by medical practitioners, since it requires less training data and fewer parameters to configure.

## Methods

In this section, we discuss the proposed PMM for ECG data in detail. The process includes time series sampling, personalized motif discovery, and motif-based prediction using an artificial logical network.

### Time Series Sampling

We treated each patient's ECG measures as individual data; the recording is a time series. An ECG is a medical test that detects heart problems by measuring the electrical activity generated by the heart as it contracts. An ECG complex is composed of different components, or waves, that represent the electrical activity in specific regions of the heart. ECG readings from healthy hearts have a characteristic shape. If the ECG reading is a different shape, that could suggest a heart problem.

During this stage, there is an important parameter, *t*, which represents the training ratio (0<*t*<1) and affects the sampling process. Before starting to discover motifs, each individual patient's ECG time series was sampled as individual training data. *L* represents the length of each patient's ECG time series. Based on the value of the training ratio *t*, we used Equation 1 to calculate the length of time series *S* we should take from the whole ECG reading of each patient:

*S* = *L* x *t*  **(1)**

As calculated, we took the first *S* length of time series from the ECG readings of each individual patient as the individual training sample data to generate the personalized motifs. During sampling, we started from the first point of the patient's ECG time series and sampled until the length of sample reached the expected sample size *S*. We then divided the sampled ECG data into *M* subsequences based on Equation 2 and each subsequence was regarded as a pattern unit.

*M* = *S*/180 **(2)**

**Figure 1:**
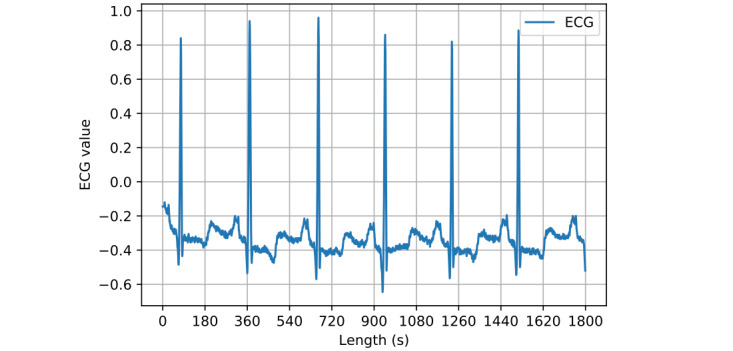
ECG sample. ECG: electrocardiogram.

In Equation 2, the number 180 is the sampling rate of the ECG recording device. It partitions the ECG into heartbeats with sufficient precision of intervals for heart rate variability analysis [34]. Figure 1 shows an ECG sample with 1800 points from one of the patients.

### Personalized Motif Discovery

After sampling, we discovered personalized motifs from the *M* subsequences sampled from an individual patient. For this model design, we needed to consider two major parameters that could affect the performance of motif discovery: (1) *r*, the time series similarity threshold and (2) *k*, the number of motifs.

**Figure 2:**
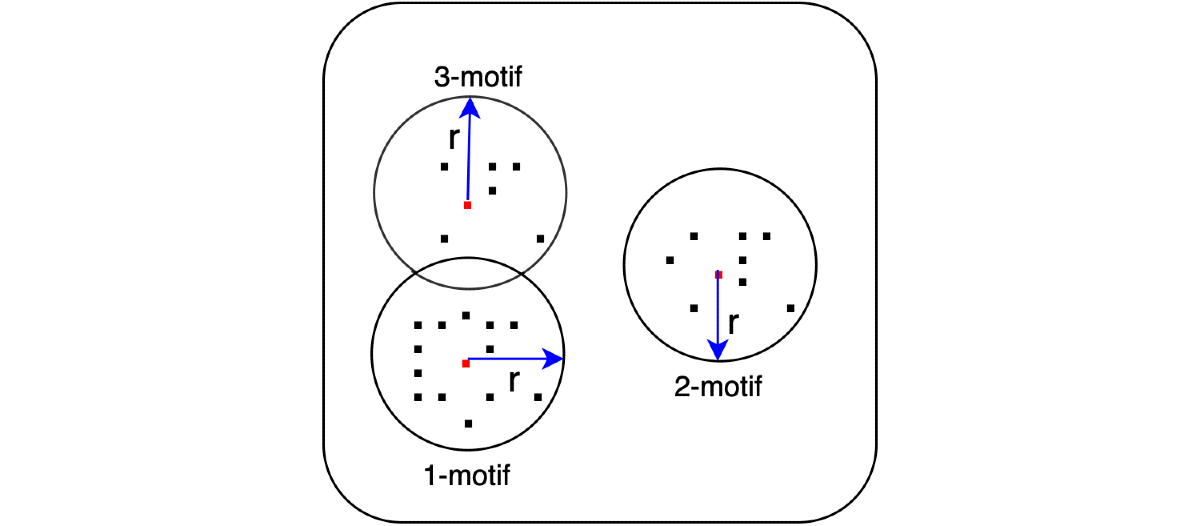
Subsequence motifs (k=3).

We calculated all Euclidean distances between each subsequence and generated motif candidates. In each motif circle/cluster, the distances from the central subsequence to other subsequences that belong to the same motif circle must be less than *r* and all motif circles cannot share the same subsequence, as proposed by a previous paper [35] and as shown in Figure 1. In Figure 2, each black dot represents each subsequence and each red dot represents the central subsequence in that motif circle. We supposed *k*=3, as we can see 1-motif has the most subsequences, 2-motif has the second most, and 3-motif has the fewest subsequences. All motif circles have the same radius *r*. The distance between the red central subsequence of 1-motif and the red central subsequence of 2-motif is more than *2r*, and these naturally do not share the same subsequence. However, the distance between the central subsequence of 1-motif and the central subsequence of 3-motif is less than *2r*, but they do not share the same subsequence, which is allowable during motif discovery. In this example, the 3 red central subsequences are the 3 extracted motifs.

Based on this strategy of motif discovery, we generated all motif circle candidates from the *M* subsequences. Based on the parameter *k,* which is the number of motifs, we only kept the first *k* motif circles and used the central subsequences of all these *k* motif circles as our extracted heartbeat motifs. From this stage, we obtained *k* personalized motifs for each individual patient.

### Motif-Based Prediction Using an Artificial Logical Network

**Figure 3:**
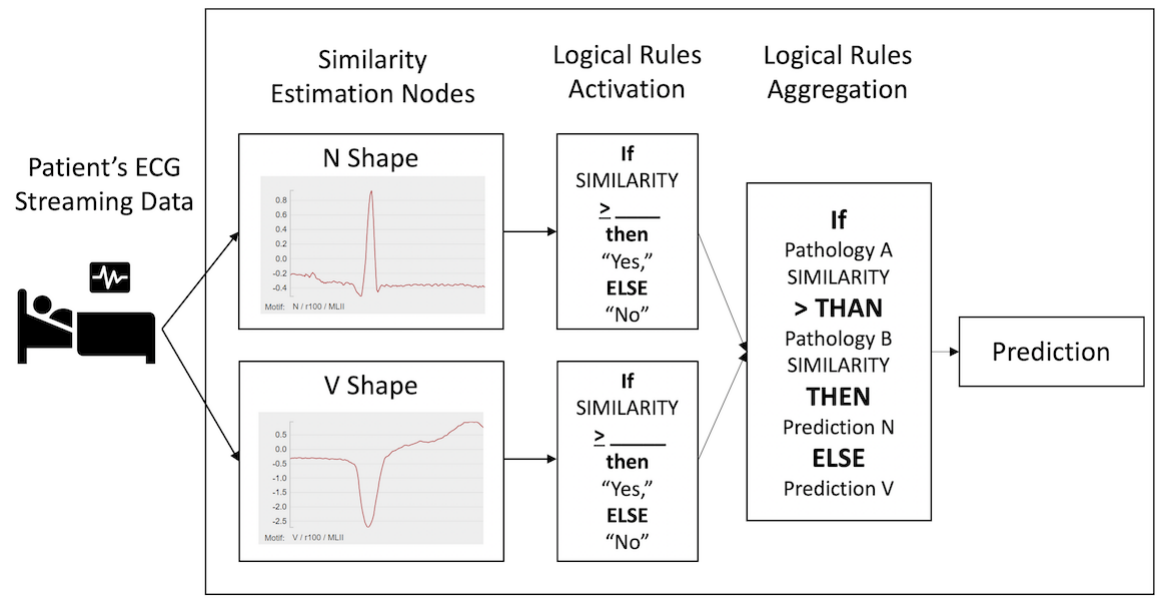
Motif-based prediction using an artificial logical network.

We used the generated *k* personalized motifs to predict the rest of the ECG readings for each corresponding patient. For example, suppose we have two types of heartbeats (N and V) and need to predict which type (N or V) the test subsequence belongs to. Following the personalized motif discovery, we generated *k* of N motifs and *k* of V motifs from the sampled subsequences of each individual patient. The generated N and V motifs were organized into an artificial logical network where each N and V motif is a dedicated evaluation node, as shown in Figure 3. For the remaining subsequences of that patient, we then tested each subsequence by comparing its distance to all N motifs and V motifs in the artificial logical network. A simple logical rule was applied to select the closest one as the predicted type of test subsequence. Finally, we predicted all labels for the rest of the subsequences of that patient. If the subsequences did not meet the matching criteria of any of the N and V nodes, the logical rule identified them as new anomalies for future learning. The combination of dedicated motif comparison and logical rules allows us to easily build a prediction system.

## Results

### Benchmark Data

For this benchmark data, we used 32 patients' ECG measures; each measure contained 30 minutes of ECG readings from each patient. In the data set, there were 5 different types of heartbeats (V, N, A, F, and S). The V pathology is expected to be morphologically different than the normal N. To detect the A pathology, we needed to monitor the frequency of the heartbeats and identify the heartbeats that appeared faster than expected. The F pathology is also expected to have a different morphology than the normal N. The S pathology is related to heart rhythm abnormalities that may not drastically change the morphology, but its occurrence is out of rhythm. Before training and testing the models, we removed the “noisy” heartbeats and only kept N heartbeats and V heartbeats as the two labels the models would identify.

### Baseline Models

We evaluated and compared the following 3 models:

1. Generalized Monitoring Model 1 (GMM1): Based on the training ratio *t*, we took the first *t* percent samples from each of 32 patients and combined the samples from those 32 patients together to extract the *k* of N motifs and *k* of V motifs as the N and V heartbeat motifs. During the testing stage, we applied the extracted N motifs and V motifs to the rest of the ECG readings for each patient and predicted the label (N or V).
2. Generalized Monitoring Model 2 (GMM2): We first extracted all N heartbeats and V heartbeats from all 32 patients. Based on the training ratio *t*, we then randomly extracted *t* percent samples from all N heartbeats and *t* percent samples from all V heartbeats. Next, we extracted the *k* of N motifs from the N heartbeat samples and the *k* of V motifs from the V heartbeat samples. The testing was the same as for GMM1. The main difference between GMM1 and GMM2 is how the training data were sampled.
3. Our proposed Personalized Monitoring Model (PMM): In this personalized model, based on the training ratio *t*, we only extracted the first *t* percent of N heartbeats and the first *t* percent of V heartbeats from an individual patient and then generated a set of personalized *k* of N motifs and *k* of V motifs to test the rest of the ECG readings of that patient. The main strategy here was to individually extract personalized motifs for the current patient and use those extracted motifs to predict the rest of readings for that patient.

There are 3 major parameters that could affect the models' performance, which are compared in detail in this section: (1) *r*, the time series similarity threshold, (2) *k*, the number of motifs, and (3) *t*, the training ratio.

To compare the models and determine which model is the best, we evaluated them based on the following 2 factors:

1. Accuracy: We needed to get an estimate of how accurate each model is on unseen/test data. For all tested heartbeats, we have the corresponding ground-truth information, which is the original label. By comparing the predicted label with the original label, we can calculate how many heartbeats were correctly predicted. Therefore, accuracy is calculated by taking the number of heartbeats predicted correctly and dividing it by the number of all heartbeats tested.
2. Running time: To obtain the final prediction results faster, we also needed to guarantee the chosen model was the fastest, including training and testing time.

### Effectiveness Evaluation

Considering the 3 main parameters *r*, *k,* and *t*, we designed the effectiveness evaluation by adjusting the values of these 3 parameters and seeing how the performance of each model changed. In figures, we used the corresponding capital letters *R*, *K*, and *T* of *r*, *k*, and *t* for clear representation.

**Figure 4:**
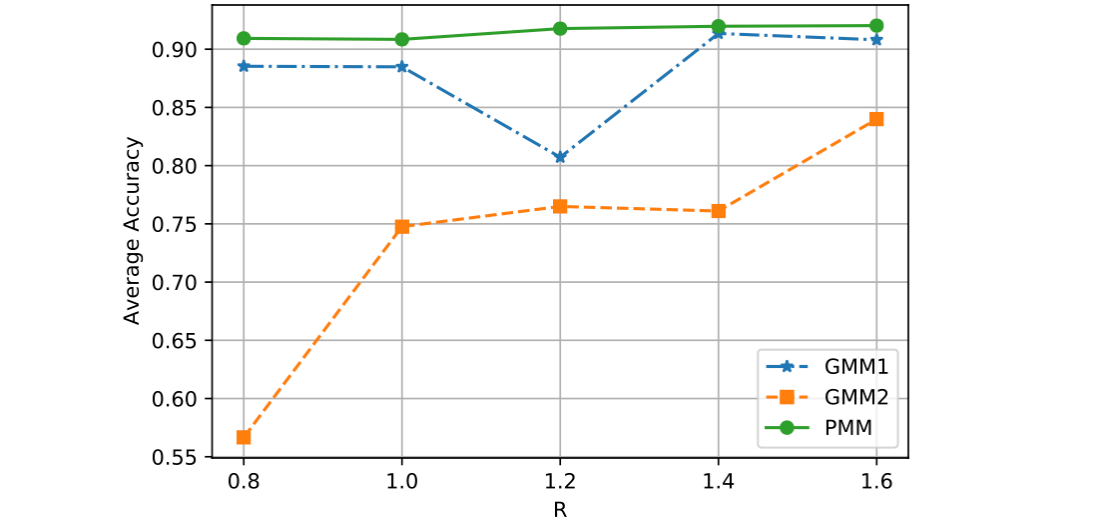
Performance comparison with different R (similarity thresholds).

First, we adjusted the similarity threshold *R* values from 0.8 to 1.6, by steps of 0.2, and observed how the average performance changed for each model. The average accuracy was calculated based on the sum accuracy across all 32 patients. Figure 4 shows 3 curves, each representing the average accuracy change of each model when *R* was increased from 0.8 to 1.6. The green line represents the PMM, which performed the best among the 3 models in terms of stability and accuracy.

**Figure 5:**
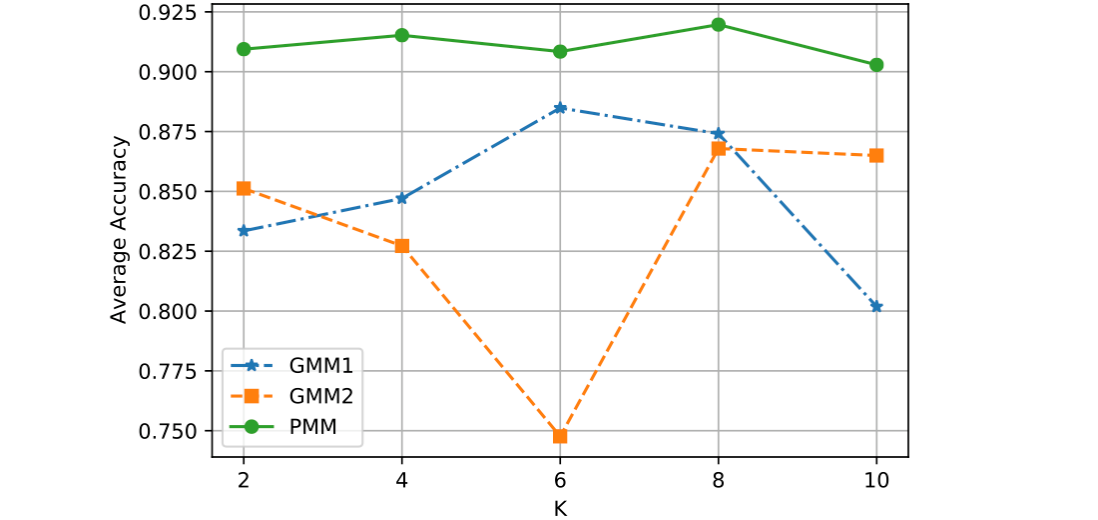
Performance comparison with different K (number of motifs).

Second, we adjusted the *K* values from 2 to 10, by steps of 2, and observed how average performance changed for each model. The PMM still performed the best, even as the *K* varied (Figure 5).

**Figure 6:**
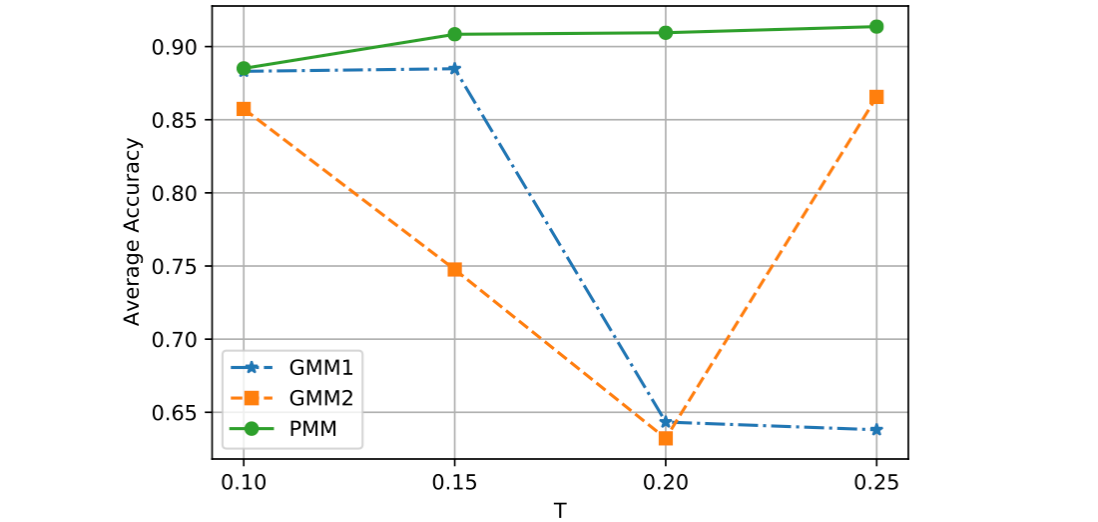
Performance comparison with different T (training ratios).

Lastly, we adjusted the training ratio *T* from 0.1 to 0.25, by steps of 0.05. Generally, more training samples result in more accurate prediction; although this is not what we observed with GMM1 and GMM2, it did apply to the PMM (Figure 6). From the curve comparison, we can see the PMM significantly outperformed GMM1 and GMM2.

*T* is the training ratio and could affect model performance more than *R* and *K* in most cases. Hence, we showed the detailed performance result for each patient as determined by the 3 models based on two sets of values of *R* and *K*: (1) *R*=0.8 and *K*=2 and (2) *R*=1 and *K*=6.

**Figure 7:**
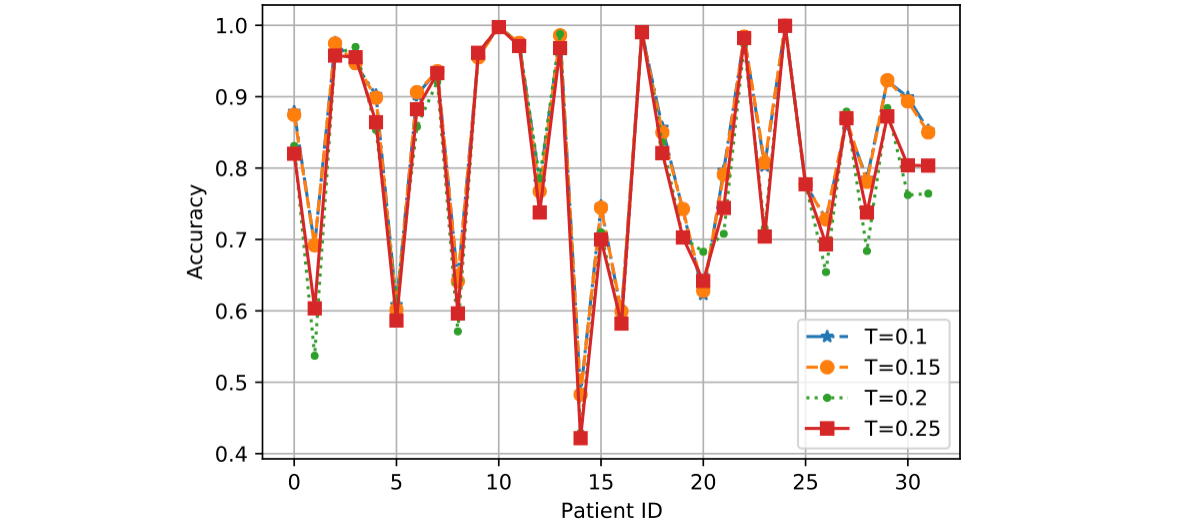
GMM1 results when R=0.8 and K=2: performance comparison on each patient with different T. GMM1: Generalized Monitoring Model 1.

**Figure 8:**
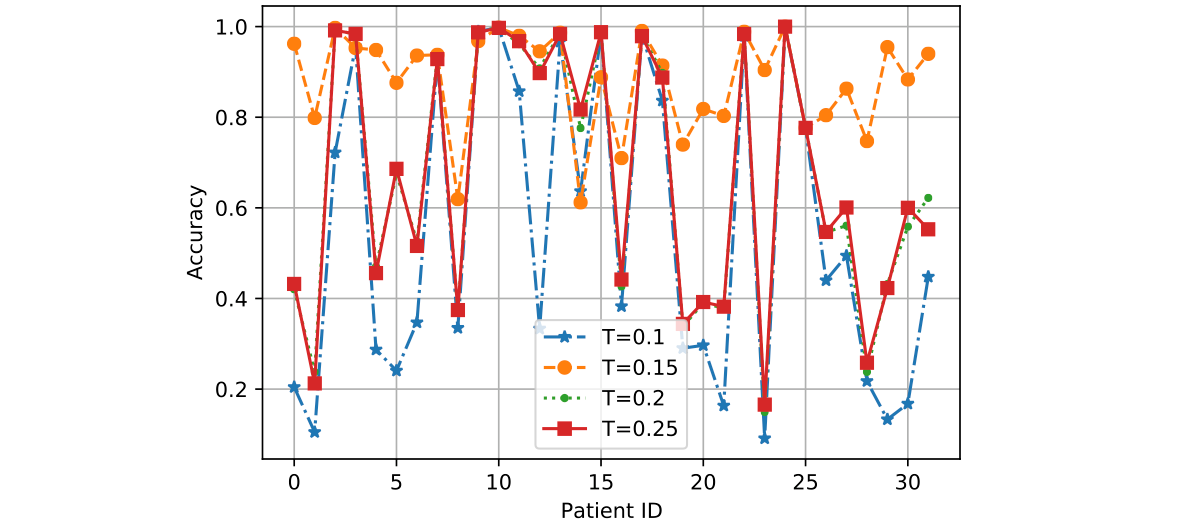
GMM2 results when R=0.8 and K=2: performance comparison on each patient with different T. GMM2: Generalized Monitoring Model 2.

**Figure 9:**
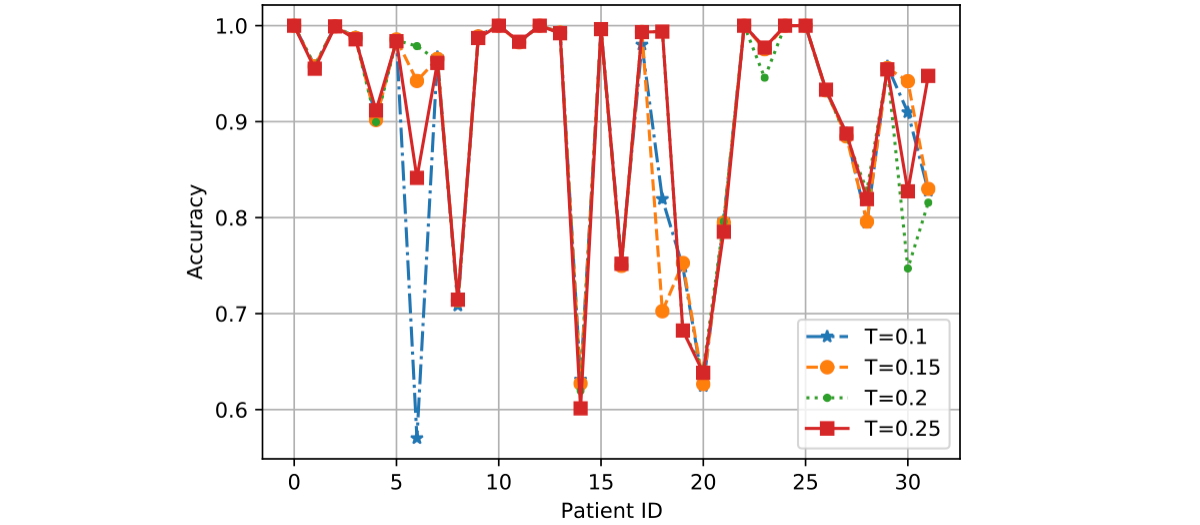
PMM results when R=0.8 and K=2: performance comparison on each patient with different T. PMM: Personalized Monitoring Model.

**Figure 10:**
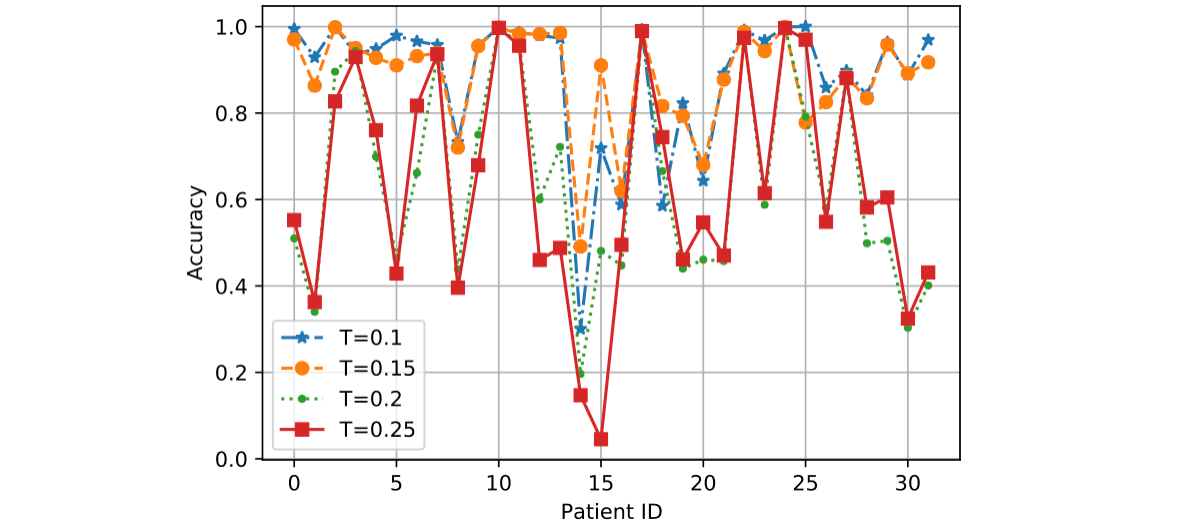
GMM1 results when R=1 and K=6: performance comparison on each patient with different T. GMM1: Generalized Monitoring Model 1.

**Figure 11:**
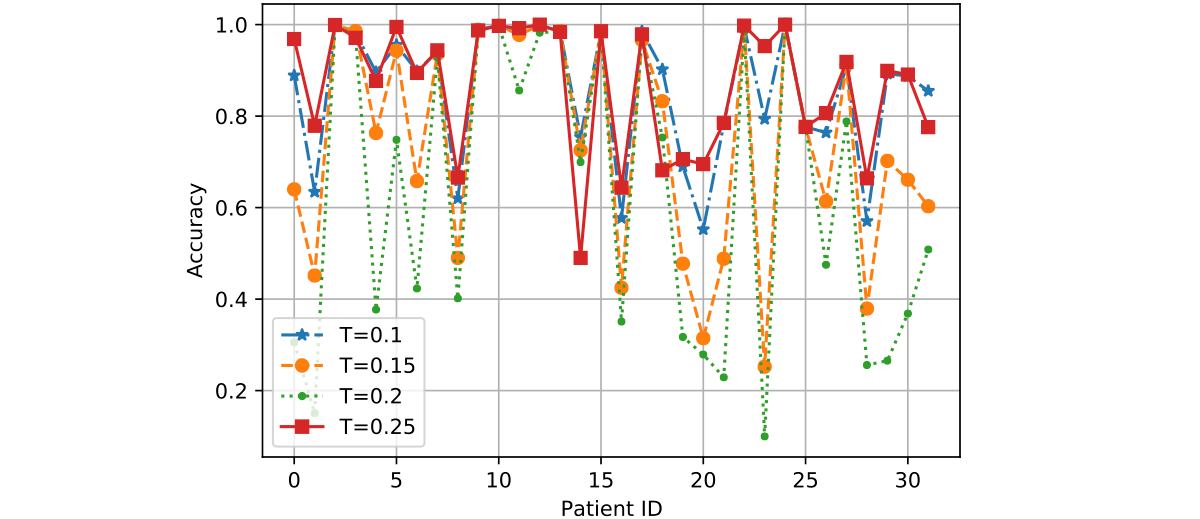
GMM2 results when R=1 and K=6: performance comparison on each patient with different T. GMM2: Generalized Monitoring Model 2.

**Figure 12:**
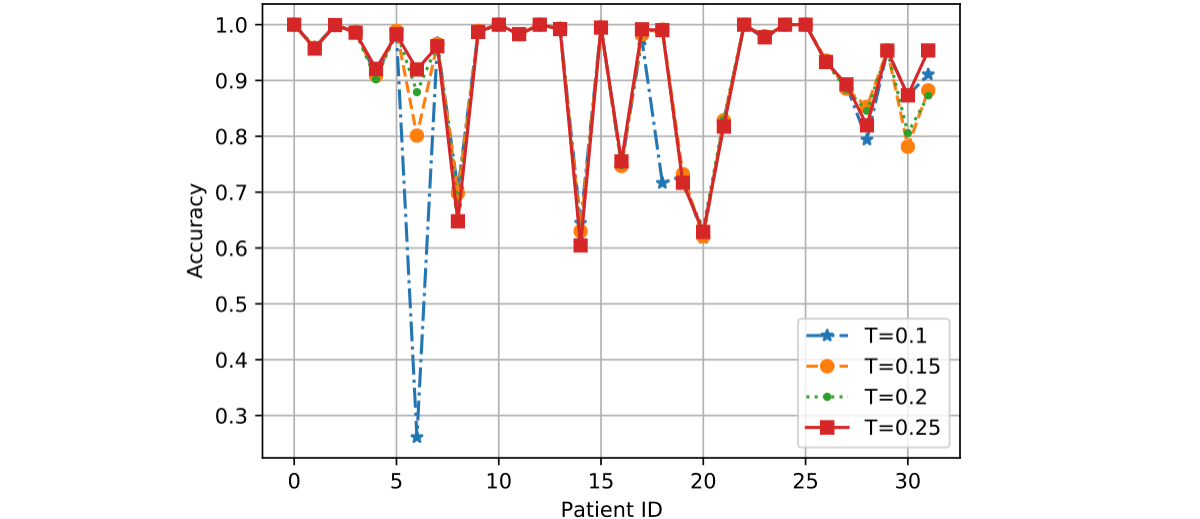
PMM results when R=1 and K=6: performance comparison on each patient with different T. PMM: Personalized Monitoring Model.

**Figure 13:**
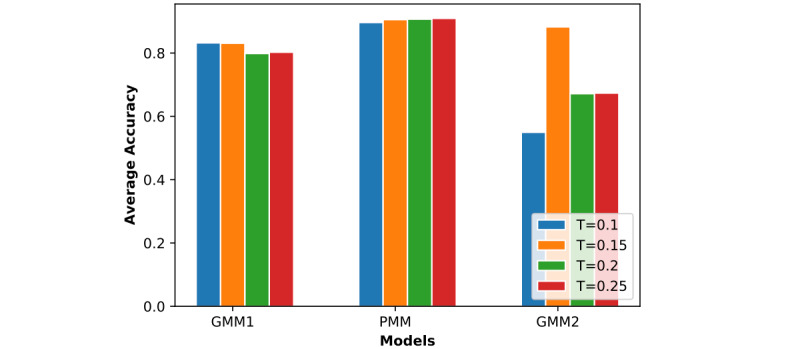
Results from all models when R=0.8 and K=2: average performance comparison with different T. GMM1: Generalized Monitoring Model 1; GMM2: Generalized Monitoring Model 2; PMM: Personalized Monitoring Model.

**Figure 14:**
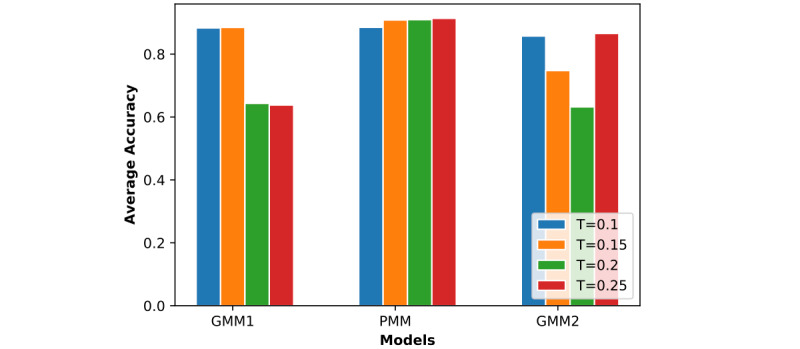
Results from all models when R=1 and K=6: average performance comparison with different T. GMM1: Generalized Monitoring Model 1; GMM2: Generalized Monitoring Model 2; PMM: Personalized Monitoring Model.

In Figures 7 to 12, we can see GMM2 fluctuated much more than GMM1 and the PMM. However, if we compare GMM1 and the PMM when *T* was increased from 0.1 to 0.25, the PMM gradually performed better on almost all patients, while GMM1 became worse as that parameter changed. In Figures 13 and 14, the two bar charts show the average accuracy of each model with different *T*. As *T* increases, GMM1’s performance becomes worse, while GMM2's performance fluctuates the most. The PMM performed the best.

### Efficiency Evaluation

**Figure 15:**
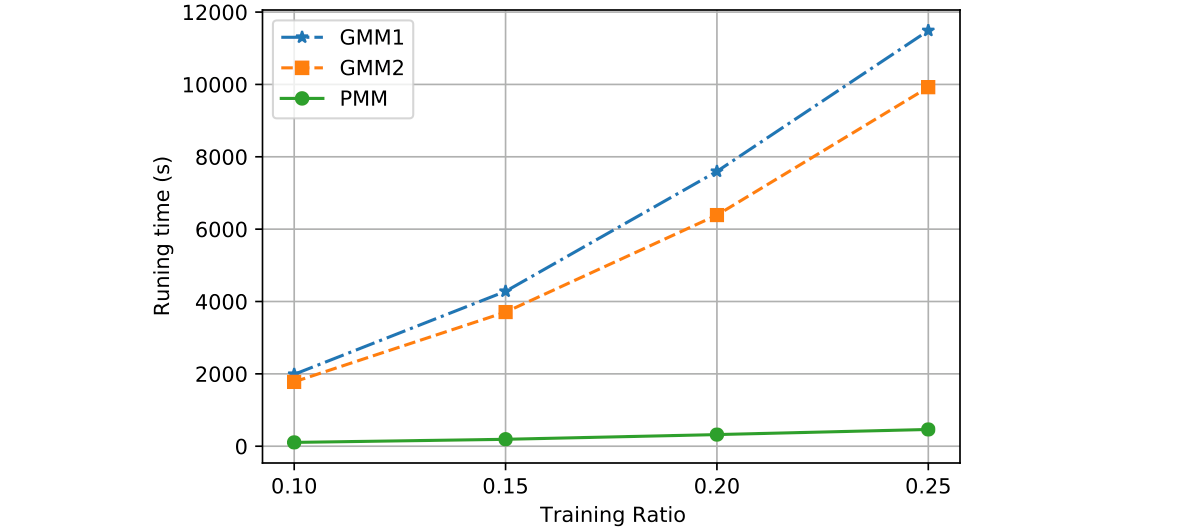
Linear comparison of time efficiency across models when R=0.8 and K=2. GMM1: Generalized Monitoring Model 1; GMM2: Generalized Monitoring Model 2; PMM: Personalized Monitoring Model.

**Figure 16:**
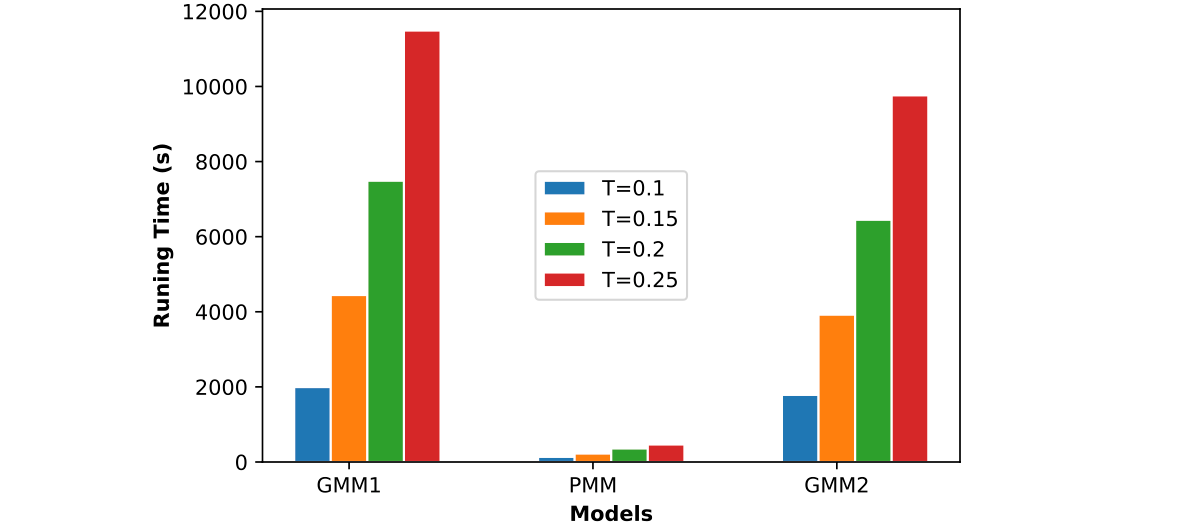
Time efficiency of all models when R=1 and K=6. GMM1: Generalized Monitoring Model 1; GMM2: Generalized Monitoring Model 2; PMM: Personalized Monitoring Model.

We evaluated the time efficiency of each model and observed which model runs the fastest. The process consists of two stages: training and testing. We considered all computation time in this evaluation, including the training and testing time. From the 3 evaluated parameters in the previous section, we know that the training ratio *T* is the one that most affects training time. Here, we adjusted the value of *T* and observed the corresponding running time of each model. Figure 15 shows the linear change in running time for each model, while Figure 16 is a bar chart of the running time. The time consumption of GMM1 and GMM2 increased almost exponentially with an increase in *T*. However, the time consumption of the PMM was linear and the lowest among the 3 models.

## Discussion

### Principal Results

According to the empirical results, the PMM performed the best in all cases in terms of prediction accuracy and time efficiency.

Figure 4 shows the average classification accuracy (y-axis) with respect to the *R* (x-axis) of all 3 models when using different similarity thresholds *R*. The PMM's performance (green line) becomes better as *R* increases from 0.8 to 1.4, and becomes stable when *R* reaches 1.6. The prediction performance of the PMM is relatively stable for similarity threshold *R*. If we compare GMM1 and GMM2, we find GMM1 outperforms GMM2. However, GMM1's performance (blue curve) was unstable with respect to *R*, and GMM2's performance (orange curve) became better as *R* increases, although the overall performance of GMM2 was worse than GMM1. This is because GMM2 extracts all N heartbeats and V heartbeats from all 32 patients for motif discovery and a greater similarity threshold allows GMM2 to aggregate more similar heartbeats for motif circles, which may help GMM2 find more representative motifs for heartbeat prediction. However, if the similarity threshold *R* is too big, it may introduce different types of heartbeats into a given motif circle, which may result in worse prediction. GMM1 takes the first *T* percent of samples from 32 patients and then extracts motifs based on the similarity distance threshold *R*. An increase in *R* may introduce more heartbeats from different patients and the extracted motifs may result in a fluctuation in accuracy. However, the PMM only extracts the first *T* percent of N samples and V samples from one patient and then generates personalized motifs to test the rest of the ECG readings of that patient. As *R* increases, more similar N heartbeats and V heartbeats will be collected for motif discovery, which could help enhance prediction if *R* is not so large that it introduces another type of heartbeat.

Figure 5 shows the average classification accuracy (y-axis) with respect to the *K* (x-axis) of all 3 models, by using different numbers of motifs *K*.

The PMM was always the best, but the GMM1 and GMM2 curves fluctuated as *K*, which represents the number of motifs, changed. A certain number of motifs can be representative and guide prediction well. However, having too many motifs may introduce unrepresentative motifs, which could hinder prediction. This is why all models fluctuated as *K* changed.

Figure 6 shows the average classification accuracy (y-axis) with respect to *T* (x-axis) on all 3 models by using different training ratios *T* from 0.1 to 0.25. As *T* increases from 0.1 to 0.25 in steps of 0.05, the PMM curve becomes higher. However, the GMM1 and GMM2 curves fluctuate a lot and are both lowest when *T* is 0.2. Generally, in machine learning, more training data should improve a model's performance. However, if more interference or noisy data is introduced into the training data, then performance will degrade. This could explain why GMM1 and GMM2 fluctuate a lot, as these models used training data from different patients and most heartbeats are unique at the patient level. As for the PMM, a larger training ratio could help improve performance because all training heartbeats are from the same patient, which avoids the introduction of interference or noisy data. As *T* changed, the PMM always outperformed GMM1 and GMM2 (Figures 7 to 14).

Based on the overall evaluation results, the PMM significantly outperformed GMM1 and GMM2 in terms of prediction accuracy.

Considering time efficiency, Figures 15 and 16 show the average running time (y-axis) with respect to the training ratio *T* (x-axis) for all 3 models by using different training ratios *T* from 0.1 to 0.25. Compared with GMM1 and GMM2, the PMM required significantly less running time. The average running time of the 3 models increased as the training ratio increased. This is because a larger training ratio results in more training data, which increases the training time accordingly. The overall average time required by GMM1 is close to that required by GMM2 and both increase almost exponentially as the training ratio increases. Therefore, the PMM has more stable and better efficiency as the training ratio increases.

### Limitations

A limitation of the PMM is that it might be hard to maintain the models for each patient. However, this limitation is not significant given that retraining the model does not take a lot of time. In addition, the cost of maintenance and computation might be lower in the future as more industries adopt personalized models.

### Conclusions

In this paper, we proposed a PMM for ECG recordings for two reasons: (1) the COVID-19 pandemic has accelerated the adoption of remote diagnosis and patient monitoring and (2) personalized care promises better outcomes, especially as it applies to digital health. Digital health care allows for continuous 24/7 care in the home environment while minimizing the risk of fatal accidents and re-admissions. For remote monitoring to gain traction at scale, several requirements must be met. First, monitoring has to be sufficiently automated with fewer false positive alarms to minimize the number of health professionals involved in monitoring. Second, it has to be quickly and easily configurable by the health care professionals themselves. Although traditional machine learning and deep learning approaches can be used in automation, they typically cannot be easily configured or adjusted by health care professionals due to a lack of modeling skills and data needed to build a personalized model. To solve these challenges, we employed a motif discovery algorithm to individually extract personalized motifs for each individual patient and used an artificial logical network for ECG signal prediction. We proposed a personalized model for faster ECG signal detection, which significantly improves the efficiency of ECG prediction; such a model could help satisfy the demand for remote monitoring services, especially during the COVID-19 pandemic. By comparing our proposed model, PMM, with two generalized monitoring models using real-world patient ECG data, we demonstrated that the PMM outperformed the generalized models in both prediction accuracy and time efficiency.

Per our discussions with clinicians, this approach can easily be deployed for outpatient monitoring as outlined below; this is the subject of a forthcoming clinical trial. A wearable 12-channel ECG monitor is sent to a patient or configured during a hospital stay. An augmented reality app or video conference is used to remotely guide the patient to accurately place the electrodes, while simultaneously testing the accuracy of the received signal. During setup, personalized motifs are automatically extracted, and the physician selects the center motifs to be used by the artificial logical network. The artificial logical network is a flexible structure that allows for learning when particular reference motifs are missing from the setup sample. For example, if samples of atrial fibrillation motifs are not recorded during setup, reference motifs from a general library can be used, or general anomaly detection can be applied, alerting a medical professional to review any anomalous occurrences. If the physician determines that the anomaly is atrial fibrillation, they can instantly push the motif to the artificial logical network. These configurations are the subject of the forthcoming clinical trial. By augmenting the expert’s knowledge with algorithmic computational power, hospital stays can be significantly reduced, and care can be delivered in the comfort of the patient’s home.

## Abbreviations

ECG: electrocardiogram
GMM: Generalized Monitoring Model
PMM: Personalized Monitoring Model
RPM: remote patient monitoring

## References

[1] Mann H (1920). A Method of Analyzing the Electrocardiogram. Arch Intern Med.

[2] Nattel S (2010). Sudden cardio arrest: when normal ECG variants turn lethal. Nat Med.

[3] Guo S, Han L, Liu H, Si Q, Kong D, Guo F (2016). The future of remote ECG monitoring systems. J Geriatr Cardiol.

[4] Sayadi O, Shamsollahi M, Clifford G (2010). Robust Detection of Premature Ventricular Contractions Using a Wave-Based Bayesian Framework. IEEE Trans Biomed Eng.

[5] Kropf M, Hayn D, Schreier G (2017). ECG classification based on time and frequency domain features using random forests. Computing in Cardiology (CinC).

[6] Alarsan FI, Younes M (2019). Analysis and classification of heart diseases using heartbeat features and machine learning algorithms. J Big Data.

[7] Wang H, Wu J (2017). Boosting for Real-Time Multivariate Time Series Classification.

[8] Li Q, Rajagopalan C, Clifford GD (2014). A machine learning approach to multi-level ECG signal quality classification. Comput Methods Programs Biomed.

[9] Castells F, Laguna P, Sörnmo L, Bollmann A, Roig JM (2007). Principal Component Analysis in ECG Signal Processing. EURASIP J Adv Signal Process.

[10] Monasterio V, Laguna P, MartÍnez JP (2009). Multilead Analysis of T-Wave Alternans in the ECG Using Principal Component Analysis. IEEE Trans Biomed Eng.

[11] Martis RJ, Acharya UR, Mandana K, Ray A, Chakraborty C (2012). Application of principal component analysis to ECG signals for automated diagnosis of cardiac health. Expert Systems with Applications.

[12] Kallas M, Francis C, Kanaan L, Merheb D, Honeine P, Amoud H (2012). Multi-class SVM classification combined with kernel PCA feature extraction of ECG signals.

[13] Kachuee M, Fazeli S, Sarrafzadeh M (2018). ECG heartbeat classification: A deep transferable representation.

[14] Afonso V, Tompkins W, Nguyen T, Luo S (1999). ECG beat detection using filter banks. IEEE Trans Biomed Eng.

[15] Ribeiro AH, Ribeiro MH, Paixão GMM, Oliveira DM, Gomes PR, Canazart JA, Ferreira MPS, Andersson CR, Macfarlane PW, Meira W, Schön TB, Ribeiro ALP (2020). Automatic diagnosis of the 12-lead ECG using a deep neural network. Nat Commun.

[16] Pyakillya B, Kazachenko N, Mikhailovsky N (2017). Deep Learning for ECG Classification. J Phys Conf Ser.

[17] Zhang J, Tian J, Cao Y, Yang Y, Xu X, Wen C (2019). Fine-grained ECG classification based on deep CNN and online decision fusion. Comput Res Repos.

[18] Jun T, Nguyen H, Kang D, Kim D, Kim D, Kim Y ECG arrhythmia classification using a 2-D convolutional neural network. arXiv.

[19] Rajpurkar P, Hannun A, Haghpanahi M, Bourn C, Ng A Cardiologist-level arrhythmia detection with convolutional neural networks. arXiv.

[20] Tang W, Long G, Liu L, Zhou T, Jiang J, Blumenstein M Rethinking 1D-CNN for Time Series Classification: A Stronger Baseline. arXiv.

[21] Chen W, Wang S, Long G, Yao L, Sheng Q, Li X (2018). Dynamic illness severity prediction via multi-task RNNs for intensive care unit.

[22] Ismail Fawaz H, Forestier G, Weber J, Idoumghar L, Muller P (2019). Deep learning for time series classification: a review. Data Min Knowl Disc.

[23] Chartrand G, Cheng PM, Vorontsov E, Drozdzal M, Turcotte S, Pal CJ, Kadoury S, Tang A (2017). Deep Learning: A Primer for Radiologists. Radiographics.

[24] Shyu L, Wu Y, Hu W (2004). Using Wavelet Transform and Fuzzy Neural Network for VPC Detection From the Holter ECG. IEEE Trans Biomed Eng.

[25] Wang F, Casalino LP, Khullar D (2019). Deep Learning in Medicine-Promise, Progress, and Challenges. JAMA Intern Med.

[26] Xiao C, Choi E, Sun J (2018). Opportunities and challenges in developing deep learning models using electronic health records data: a systematic review. J Am Med Inform Assoc.

[27] Shameer K, Johnson KW, Glicksberg BS, Dudley JT, Sengupta PP (2018). Machine learning in cardiovascular medicine: are we there yet?. Heart.

[28] Wiens J, Shenoy E (2018). Machine Learning for Healthcare: On the Verge of a Major Shift in Healthcare Epidemiology. Clin Infect Dis.

[29] Miotto R, Wang F, Wang S, Jiang X, Dudley J (2018). Deep learning for healthcare: review, opportunities and challenges. Brief Bioinform.

[30] Chen D, Liu S, Kingsbury P, Sohn S, Storlie CB, Habermann EB, Naessens JM, Larson DW, Liu H (2019). Deep learning and alternative learning strategies for retrospective real-world clinical data. NPJ Digit Med.

[31] Jambukia S, Dabhi V, Prajapati H (2015). Classification of ECG signals using machine learning techniques: A survey.

[32] Pathinarupothi R, Rangan E (2016). Discovering vital trends for personalized healthcare delivery. Proceedings of the 2016 ACM International Joint Conference on Pervasive and Ubiquitous Computing: Adjunct.

[33] Mueen A, Chavoshi N (2014). Enumeration of time series motifs of all lengths. Knowl Inf Syst.

[34] Kwon O, Jeong J, Kim HB, Kwon IH, Park SY, Kim JE, Choi Y (2018). Electrocardiogram Sampling Frequency Range Acceptable for Heart Rate Variability Analysis. Healthc Inform Res.

[35] Chi L, Chi H, Feng Y, Wang S, Cao Z (2011). Comprehensive and efficient discovery of time series motifs. J Zhejiang Univ - Sci C.

